# Supplementary material for: Compounds Containing 2,3‐Bis(phenylamino) Quinoxaline Exhibit Activity Against Methicillin‐Resistant Staphylococcus aureus, Enterococcus faecalis, and Their Biofilms
Source: Microbiologyopen. 2024 Dec 12;13(6):e011. doi: 10.1002/mbo3.70011 (PMC11635387; doi:10.1002/mbo3.70011)
Supplement: Supplementary file 4 — Supporting information. [file MBO3-13-e011-s003.docx]

**Appendix**

**Table S1.** Summary of the workflow of the study described in the paper arranged by compound (**Table S1A**) and by strain (**Table S1B**).

**Table S2.** List of the 19 clinically relevant strains used during Phase 1 of the study (**Table S2A**) and the minimum inhibitory concentrations (MICs, mg/l) of the six compounds tested (**Table S2B**).

**Table S3.** Minimum inhibitory concentrations (MICs, mg/l) of two selected compounds and four comparative agents against a panel of *S. aureus*, *E. faecalis* and *E. faecium* (**Table S3A**) interpreted based on the current EUCAST breakpoint (summarised in **Table S3B**).
